# Postnatal regulation of MAMDC4 in the porcine intestinal epithelium is influenced by bacterial colonization

**DOI:** 10.14814/phy2.13018

**Published:** 2016-11-08

**Authors:** Alex J. Pasternak, Glenn M. Hamonic, Andrew Van Kessel, Heather L. Wilson

**Affiliations:** ^1^ Vaccine and Infectious Disease OrganizationInternational Vaccine Centre (VIDO‐InterVac)University of SaskatchewanSaskatoonSaskatchewanCanada; ^2^ Department of Animal and Poultry ScienceUniversity of SaskatchewanSaskatoonSaskatchewanCanada

**Keywords:** Commensal flora, epithelium, gastrointestinal, host–microbial interaction, MAMDC4, neonate

## Abstract

The MAM domain‐containing 4 (MAMDC4) protein is associated with the unique endocytotic mechanism observed in the intestine of mammals during the immediate postnatal period. Transcriptional expression of MAMDC4 was substantially upregulated at birth in both the piglet jejunum and ileum and its expression decreases after birth. The protein was found localized specifically to the apical region of the luminal epithelium, however, MAMDC4 protein expression was lost at day 10 and 15 in the jejunum and ileum, respectively, and was not associated with “fetal” enterocyte replacement. Although spatial variation in the subcellular localization of Claudin 1 (CLDN1) was noted at day 3, the loss of MAMDC4 at later stages of development did not appear to have any effect on the tight junction structure. Germ‐free (GF) piglets and piglets whose gastrointestinal flora consists exclusively of *Escherichia coli* (EC) or *Lactobacillus fermentum* (LF) maintained MAMDC4 protein expression to 14 days of age in distal regions of the small intestine whereas those with conventionalized intestinal flora (CV) showed no MAMDC4 protein at this age. MAMDC4 protein expression was most pronounced in the LF and GF colonized piglets which showed staining in the epithelial cells at 75% and 95% of the length of the small intestine, respectively, which matched that of the newborn. In contrast, EC animals showed only a low abundance at these regions as well as a discontinuous staining pattern. Collectively these results suggest that maturation of MAMDC4 expression in the porcine epithelium occurs more rapidly than what is reported in previously studied rodent species. Furthermore, intestinal bacterial colonization is a major regulator of MAMDC4 in a manner specific to bacterial species and independent of enterocyte turnover.

## Introduction

During the postnatal period, the gastrointestinal tract undergoes rapid growth and adaptation in order to assume its role in nutrient assimilation and host defense. During this acute period, the intestine is colonized by an increasingly complex microbiota which impacts intestinal development and physiology. In nearly all mammals, a major postnatal adaptation of the gastrointestinal tract is the transient absorption of specific colostral‐derived molecules across the intestinal barrier. Unlike rodents and humans, the restrictive nature of the epitheliochorial placenta found in swine results in piglets being born both hypoproteinemic (Lecce and Matrone 1960) and immunologically naive (Nechvatalova et al. 2011) which exacerbates this requirement for absorption of antibodies and proteins in the perinatal period. The gut of the newborn is therefore uniquely designed to be “semi‐permeable” to macromolecules for a limited time to allow maternal antibodies and other bioactive compounds to traverse the gut wall in an intact form (Salmon 2012). Some aspects of this process such as the uptake of maternal antibody through the FCGRT receptor (Salmon et al. 2009) are well established but other aspects such as passive paracellular transport (Svendsen et al. 1986) and pinocytosis though the apical canalicular system (Skrzypek et al. 2007) are not understood in their entirety. Our research in pigs shows that localization of tight junction proteins are not conserved between adults and neonates (Pasternak et al. 2015) and these differences in tight junction localization should be studied further to determine if they impact gut permeability.

One particular protein which would appear to be of great importance in the neonatal gut has a number of aliases including Apical Edosomal Glycoprotein (AEGP), Endotubin (ET), and Apical Early Endosomal Glycoprotein (AEEGP) but whose HUGO Gene Nomenclature Committee name is meprin, A‐5 protein, and receptor protein‐tyrosine phosphatase mu (MAM) domain‐containing 4 (MAMDC4). Initially identified in the membrane fraction of the ileum from a suckling rat (Wilson et al. 1987), MAMDC4 specifically localized to the apical aspect of the luminal epithelium through a targeting signal located in its cytoplasmic domain (Gokay et al. 2001). Electron microscopy of immunocytochemistry further narrowed protein localization to the endosomal vesicle membrane, the luminal side of endosomal tubule membranes, and the apical endocytic pits of enterocytes (Wilson et al. 1987). In vitro*,* MAMDC4 has been shown to be involved in localizing tight junction proteins such as Claudin 1 and Occludin to the paracellular membrane. Because the functional domain MAM is thought to play a role in protein binding, cell–cell interactions or adhesion, we hypothesize that MAMDC4 plays a critical role in transepithelial transport of specific colostral components in a manner akin to that observed for FCGRT. Perhaps most telling about its importance in the early intestine, MAMDC4 is found exclusively in the gastrointestinal tract of the neonate and it is entirely absent from the adult gut (Wilson et al. 1987). Although the true role of MAMDC4 protein in the physiology of the early intestine remains unknown, current data suggest it is critically required for either early function or adaptation.

To date, research on MAMDC4 has been conducted primarily in small animal models, such as rats and rabbits, and in cell culture systems. While the gene for MAMDC4 and those of its paralogs have been identified in the porcine genome, we have as yet found no studies which examine its corresponding gene and protein expression patterns in the intestine of the neonatal pig. To better understand the function and regulation of MAMDC4 in the neonatal intestine, we have characterized its temporal and spatial distribution in the neonatal pig. In addition, we have examined its association with tight junction structure and the association with establishment of the postnatal microbiota.

## Materials and Methods

### Animal use and sample collection

This work was approved by the University of Saskatchewan's Animal Research Ethics Board, and adhered to the Canadian Council on Animal Care guidelines for humane animal use. Conventionally raised Landrace cross piglets were obtained from the Prairie Swine Centre, Inc. (PSCI), Saskatoon, SK, Canada. Postnatal tissue samples were collected from conventionally raised piglets at Day 1 (*n* = 5), Day 3 (*n* = 3), Day 5 (*n* = 3), Day 10 (*n* = 3) and Day 15 (*n* = 3) relative to observed farrowing date, along with postweaning juvenile pigs at 6 weeks of age (*n* = 5). Each of the animals used was acquired from the litter of a different sow. Because swine are considerably more out‐bred that other research animal species, the number of animals was deemed sufficient to demonstrate the temporal dynamics across this developmental time course. Animals were euthanized via captive bolt followed by exsanguination. A 5‐cm segment of jejunum (Peyer's patch‐free) and ileum were excised, and immediately snap‐frozen in liquid nitrogen prior to storage at −80°C. Another section of tissue was fixed in 10% buffered formalin (Sigma‐Aldrich, Oakville, ON, Canada) for 36–48 h. Tissue processing was carried out in a Tissue Tek VIP6 vacuum processor (Torrence, CA) using standard methods and samples were subsequently embedded in paraffin.

In the current study, we perform immunohistofluorescent analysis on tissues taken from discrete regions of the gut from 14 day old gnotobiotic piglets. The following is a brief description of the published experiments where‐in we generated and confirmed the gnotobiotic status of these piglets (Shirkey et al. 2006; Willing and Van Kessel 2007). Briefly, 16 germ‐free piglets (>800 g) were delivered by caesarean section, transferred to sterile isolators and provided with irradiated colostrum. At 24 h of age, piglets were allocated to one of 4 treatment groups (*n* = 4/treatment), including remaining germ‐free (GF), being colonized exclusively with either nonpathogenic *Escherichia coli* (EC) or with *Lactobacillus fermentum* (LF), or establishing conventional flora by feeding piglets fecal matter obtained from a clinically healthy sow (CV). EC and LF were isolated from the cecum of a healthy pig and two mls of 10^8^ and 10^9^ CFU/mL of LF and EC, respectively, were added to the sterile colostrum and orally administered to the piglets 1 day postpartum (Willing and Van Kessel 2007). The gnotobiotic status of the piglets was extensively characterized throughout the course of the experiment via periodic culture of perianal swabs and the final germ‐free and mono‐associated status of the animals was verified following necropsy through the culture of digesta collected under sterile conditions from both the ileum and cecum (Shirkey et al. 2006; Willing and Van Kessel 2007).

Intestinal histology samples from all four treatment groups were subsequently obtained at necropsy performed when piglets were 14 days of age. The small intestine was dissected from the mesentery, measured, and small histological samples (1–2 cm) were taken at 25%, 50%, 75%, and 95% relative to its total length as detailed in our earlier reports in Shirkey et al. (2006) and Willing and Van Kessel (2007).

### Immunohistofluorescence

Immunohistofluorescence was conducted as previously described in Pasternak et al. (2015). Briefly, tissue sections were deparaffinized in xylene (Sigma‐Aldrich) and rehydrated to distilled water in decreasing concentrations of ethanol. Heat‐induced antigen retrieval was carried out in Tris‐EDTA buffer (10 mmol/L Tris, 1 mmol/L EDTA Solution, 0.05% Tween 20, pH 9.0; Sigma‐Aldrich) for 30 min at 90°C. Slides were blocked for 3 h at room temperature in 5% (w/v) skim milk in Tris‐buffered saline (TBS) and then incubated overnight at 4°C with either a 1:500 dilution of rabbit anti‐MAMDC4 (Novus 35320002) or a 1:100 dilution of rabbit anti‐CLDN1 (Abcam, ab15098, Cambridge, MA) or equivalent concentrations of a rabbit isotype control, in incubation buffer (1% BSA, 1% horse serum, 0.5% Triton X‐100 in PBS; Sigma‐Aldrich). Slides were then washed three time in PBS and incubated with an Alexa555‐conjugated goat anti‐rabbit antibody (1:500 dilution) (Abcam, ab150082) in incubation buffer at 4°C for 4 h. Slides were again washed three times in PBS, before counter staining in 1 μg/mL 4′,6‐diamidino‐2‐phenylindole, dilactate (DAPI) (Life Technologies, Burlington, ON, Canada) in methanol and cover slipping with Mowiol. Standard fluorescent imaging of intestinal villi was carried out, using an Axiovert 200M with a 20× and 63× neoFluor objective (Zeiss, Oberkochen, Germany). Staining was conducted on a minimum of two nonconsecutive sections and a minimum of three villi imaged per sample. Confocal Images were generated, using a TCS SP5 scanning confocal microscope (Leica Microsystems, Wetzlar, Germany), equipped with a 63× oil immersion objective and utilizing the 405 nm (UV) and 561 nm laser lines. Images of the complete intestinal cross section were produced, using the tiling functions and subsequently aligned and stitched using the LS ASF software (Leica, Leica Microsystems, Wetzlar, Germany).

### qPCR

Previously frozen, individual tissue segments were ground to a fine powder under liquid nitrogen using mortar and pestle. Total RNA was isolated from 50 mg of powdered tissue using 1 mL of Trizol (Life Technologies) by double precipitation before the removal of DNA using the Turbo DNAfree Kit (Life Technologies). The concentration and purity were evaluated via NanoDrop (Thermo Fisher Scientific, Waltham, MA) and RNA integrity was verified, using a denaturing agarose gel, here cDNA was generated from 2 μg of total RNA, using the High Capacity cDNA Reverse Transcription Kit (LifeTechnologies) as per the supplied protocol. Real‐time PCR was then conducted in duplicate, using 20 ng equivalent cDNA and SYBR green mastermix (Kapa Biosystems, Wilmington, MA) on a Step One Plus (LifeTechnologies) thermocycler. Target‐specific primers were created using the available NCBI reference sequences, and where possible designed to span exon–exon junctions as identified, using the BLAT against the Sscrofa10.2 genome (Kent 2002). Table 1 indicates the targets gene names, the melting point of their primers and the primer sequences for 6 housekeeping genes and genes of interest.

**Table 1:** Real time PCR primer sequences for porcine MAMDC4 and associated housekeeping genes. Annealing temperatures determined empirically

Target	Tm (°C)	Forward	Reverse
B2MI	60	5′‐CAAGATAGTTAAGTGGGATCGAGAC‐3′	5′‐TGGTAACATCAATACGATTTCTGA‐3′
GAPDH	63	5′‐CTTCACGACCATGGAGAAGG‐3′	5′‐CCAAGCAGTTGGTGGTACAG‐3′
HMBS2	60	5′‐AGGATGGGCAACTCTACCTG ‐3′	5′‐GATGGTGGCCTGCATAGTCT‐3′
HPRT	60	5′‐GGACTTGAATCATGTTTGTG‐3′	5′‐CAGATGTTTCCAAACTCAAC‐3′
MAMDC4	60	5′‐TTCAGGGTGACCTTCTCTGG‐3′	5′‐TTGTTCTGGCAATGGTGATG‐3′
RPL19	60	5′‐AACTCCCGTCAGCAGATCC‐3′	5′‐AGTACCCTTCCGCTTACCG‐3′
SDHA	58	5′‐CTACAAGGGGCAGGTTCTGA‐3′	5′‐AAGACAACGAGGTCCAGGAG‐3′

### Western blot

Whole tissue samples were ground to a fine powder as described above, and total protein was extracted from 200 mg using RIPA buffer (50 mmol/L Tris, 150 mmol/L NaCl, 10% SDS, 1% NP‐40, 1% Deoxycholic acid (Sigma‐Aldrich, St. Louis, MO) supplemented with 1 mmol/L PMSF (Sigma). Samples were vortexed for 1 min, incubated on ice for 30 min and then sonicated for 2 min with 10 sec pulses. Samples were centrifuged for 10 min at 10,000 × *g* and protein content in the supernatant was determined by BCA (Pierce, Waltham, MA). An aliquot of 100 ng total protein was then mixed 1:1 with loading buffer, boiled, and applied to a 10% linear acrylamide gel (BioRad, Hercules, CA). Protein was transferred to a nitrocellulose membrane, blocked in 10% skim milk for 30 min and then incubated with 1:1000 rabbit anti‐MAMDC4 antibody (Novus 35320002, Minneapolis, MN) and 1:5000 mouse anti‐*β*actin (Sigma, AC‐74) overnight at 4°C. Blots were washed three times in Tris‐buffered saline with tween (TBST) and then incubated with 1:10,000 dilution of both donkey anti‐rabbit IR800CW (LI‐COR, #32213, Lincoln, NE) and goat anti‐mouse IR680LT (LI‐COR, #32220). The blots were again washed three times and then imaged, using an Odyssey CLx (LI‐COR).

### Statistical analysis

Real‐time gene expression analysis was performed, using the 2^−ΔΔCT^ method (Livak and Schmittgen 2001) using the geometric mean of 3 (ileum) and 4 (jejunum) stable housekeeping genes (Vandesompele et al. 2002) and making comparisons relative to the mature animals. Statistical analysis was conducted using a standard one‐way ANOVA, comparing each time point that of the mature (6 week old) intestine.

## Results and Discussion

### Transcriptional expression of MAMDC4

To effectively normalize gene expression, the expression profiles of 6 housekeeping genes were assessed across time in both the jejunum and ileum during the postnatal period (Table 1). Two housekeeping genes, Hydroxymethylbilane Synthase (HMBS2) and Beta‐2‐microglobulin (B2MI), were abandoned because they showed clear statistical changes over time suggesting temporal regulation during this period (data not shown). The remaining 4 genes, including Glyceraldehyde‐3‐Phosphate Dehydrogenase (GAPDH), Hypoxanthine‐guanine phosphoribosyl‐transferase (HPRT), Succinate Dehydrogenase Complex Flavoprotein Subunit A (SDHA), and L19 ribosomal protein gene (RPL19), were found to be stable over time in the jejunum and their geometric mean was used to normalize expression of MAMDC4 (data not shown). In the ileum, HPRT was significantly altered (*P* = 0.02) in time and so expression was only normalized to the remaining three housekeeping genes.

**Figure 1:**
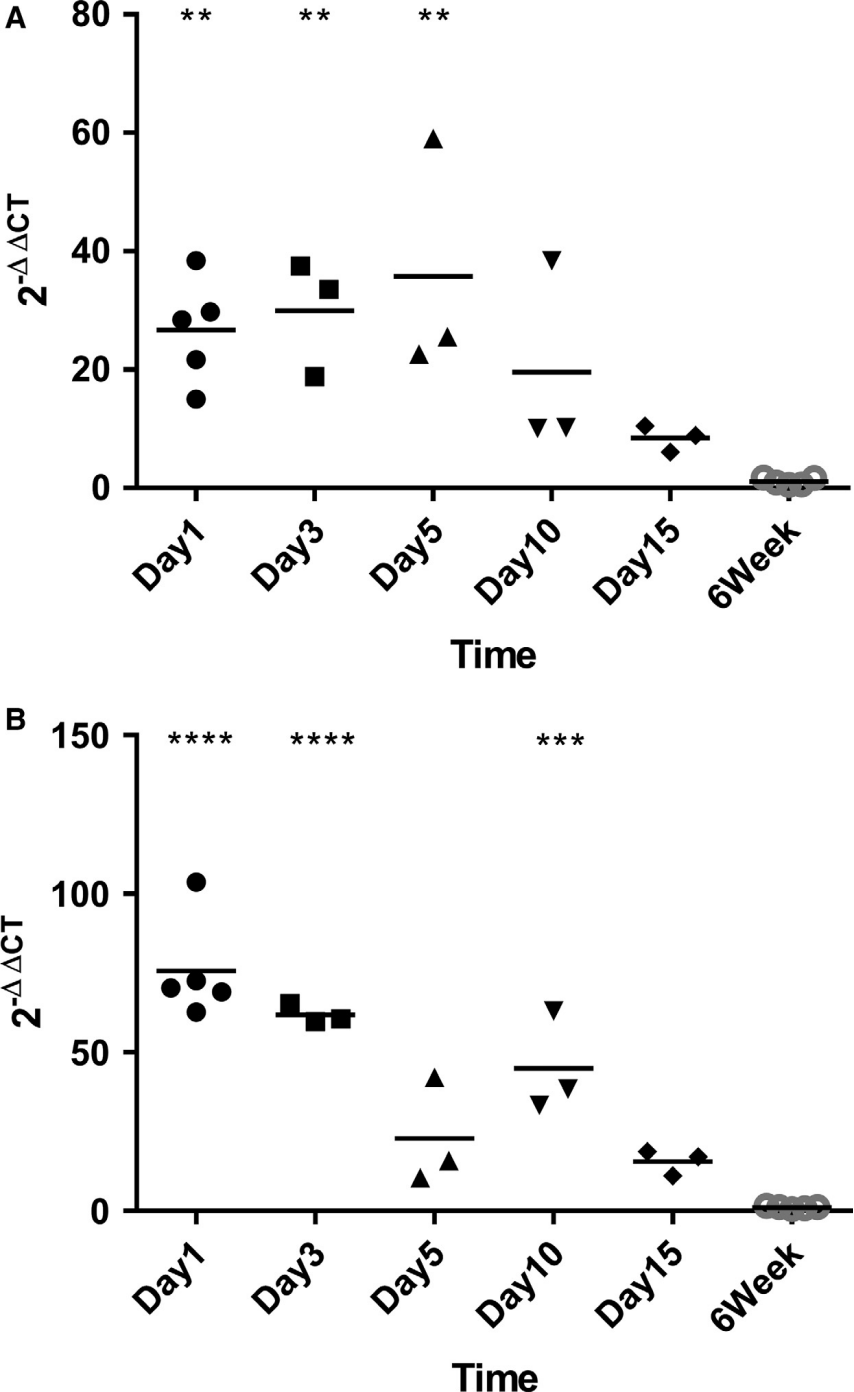
Whole tissue MAMDC4 gene expression in the (A) Jejunum and (B) Ileum during postnatal development. Results presented are normalized to the expression at 6 weeks of age within intestinal segment. Each point indicates a biological replicate.

Expression of MAMDC4 was found to be significantly elevated in the jejunum (Fig. 1A) at Day 1 (*P* < 0.01), Day 3 (*P* < 0.01) and Day 5 (*P* < 0.01) relative to the mature intestine. In the ileum (Fig. 1B), MAMDC4 gene expression was significantly elevated on Day 1 (*P* < 0.0001), Day 3 (*P* < 0.0001) and Day 10 (*P* < 0.001) relative to the mature intestine. Although both adult tissues express approximately equal quantities of the transcript, the relative abundance in the ileum on day one was found to be significantly higher than in the jejunum, at an average of 75.6 and 32.5 fold, respectively. In the jejunum, however, MAMDC4 expression showed no significant decrease out to 5 days of age (Fig. 1A), whereas in the ileum a significant decrease in transcript abundance was noted after day 3 (Fig. 1B).

Although no transcript variants of MAMDC4 have been reported in humans, the most recent build of the porcine genome (Sus Scrofa10.2/susScr3) includes five predicted transcripts (XM_013989113.1, XM_013989110.1, XM_013989106.1, XM_013989104.1, XM_013989120.1) which are similar in structure to those reported in the mouse (GRCm38.p4). The predicted variants affect the coding region and thus the resulting protein sequence but functional differences in variant proteins have not been reported in either the pig or the mouse. Our primers were designed to target a predicted universal region at the 3′ end of the sequence and thus the reported gene expression does not account for changes in variant expression.

### MAMDC4 protein expression

**Figure 2:**
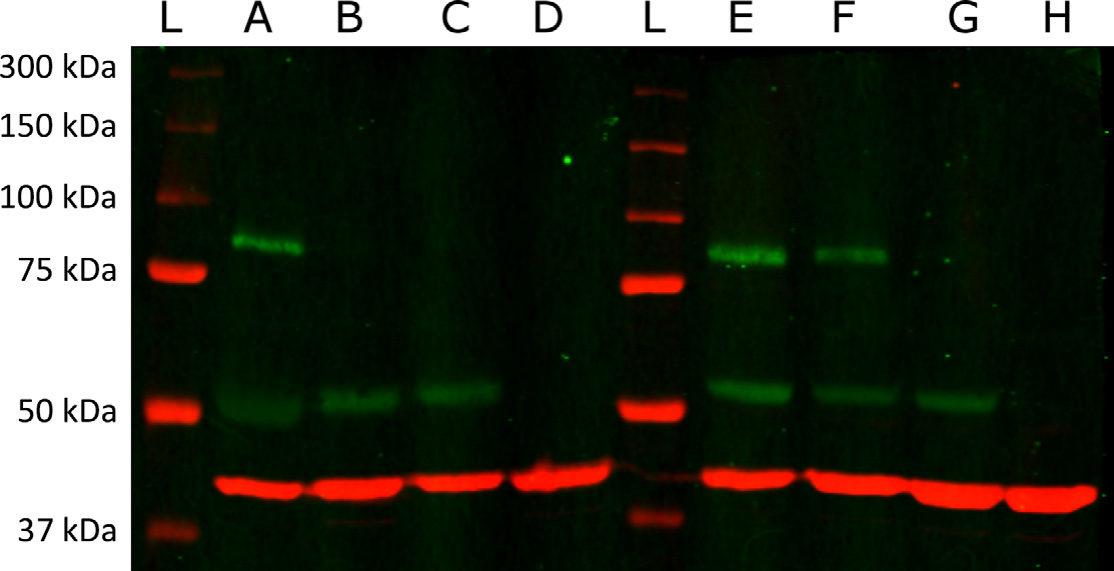
Two color denaturing Western blot, using a polyclonal antibody against MAMDC4 (Green) and *β*‐actin (Red) as a control showing representative banding pattern for whole intestinal protein from the Jejunum (A–D) and Ileum (E–H) isolated from piglets at Day 1 (A&E), Day 3 (B&F), Day 5 (C&G), and 6‐weeks (D&H) of age. Lanes‐containing molecular weight ladder marked L, with corresponding molecular weights on the left hand side.

MAMDC4 is known to localize specifically to the apical endosomal membrane in the rat ileal enterocyte where it is anchored to the cell membrane by the C‐terminus of the larger fragment (Wilson et al. 1987; Allen 1998). Using proteins processed from whole jejunum and ileum tissues from piglets 1, 3, 5 days old or 6 weeks of age, we performed Western blot analysis and we observed that 1‐day‐old piglets showed two distinct bands at 50 and 80 kDa (Fig. 2) that were detected by the rabbit anti‐MAMDC4 antibody. This dual banding pattern is consistent with the published protein structure (Allen 1998), where the protein is proteolytically cleaved to form asymmetrical heterodimers with the larger protein segment traversing the plasma membrane such that only a small portion remains in the cytosol and the smaller, N‐terminal portion of the dimer associates with the luminal face of the larger C‐terminal protein fragment. The Western blot result also confirms that the rabbit anti‐MAMDC4 polyclonal antibody is capable of reacting with both portions of the cleaved porcine protein dimer as described previously in other species (Allen 1998). This cross‐species reactivity of the polyclonal antibody is not surprising given the sequence similarity in the published protein sequence. For instance, human and swine MAMDC4 share between 71% and 73% homology depending on which full length protein variant is assessed. Much of the variation comes from a series of three sequence insertions in the pig which are absent in the rodent transcripts, which explain the increased molecular weight from 74 kDa in the mouse to 80 kDa in the pig.

In Figure 2, Lanes A, B, C, and D represent tissues obtained from jejunal gut in 1, 3, and 5 days of age as well as 6‐week old piglets, respectively (Fig. 2), whereas Lanes E, F, G, and H represent tissues obtained from ileal gut in Day 1, Day 3, Day 5 and 6‐week old piglets, respectively. Interestingly, while the lower molecular weight band can be detected in both the jejunum and ileum in tissues obtained from 1 day old piglets out to 5 days, the higher molecular weight band can only be seen for the first 24 h in the jejunum and out to 3 days in the ileum. The prolonged presence of the smaller fragment over that of its partner suggests that translation of the protein ceases prior to day 3 and 5 in the jejunum and ileum, respectively. Interestingly, this apparent loss of translation in the jejunum prior to day 3 occurs much earlier after birth than what we observed for the kinetic decrease of MAMDC4 transcript to adult levels which occurs after 5 days of age (Fig. 1A). For the ileum as well, we observe that translation of the protein appears to cease prior to day 5 but a significant decrease in transcript abundance was noted after day 3 (Fig. 1B). MAMDC4 is known to localize specifically to the apical endosomal membrane where it is anchored to the cell membrane at the C‐terminus of the larger fragment (Wilson et al. 1987). As the luminal lower molecular weight fragment requires the larger fragment to remain associated with the plasma membrane, our result may support the suggestion that the protein becomes at least partially internalized with age, consistent with electron micrographs demonstrating the presence of the molecule in the endosomal tubules in rat ileum (Wilson et al. 1987).

### Postnatal localization of MAMDC4 in the small intestine

**Figure 3:**
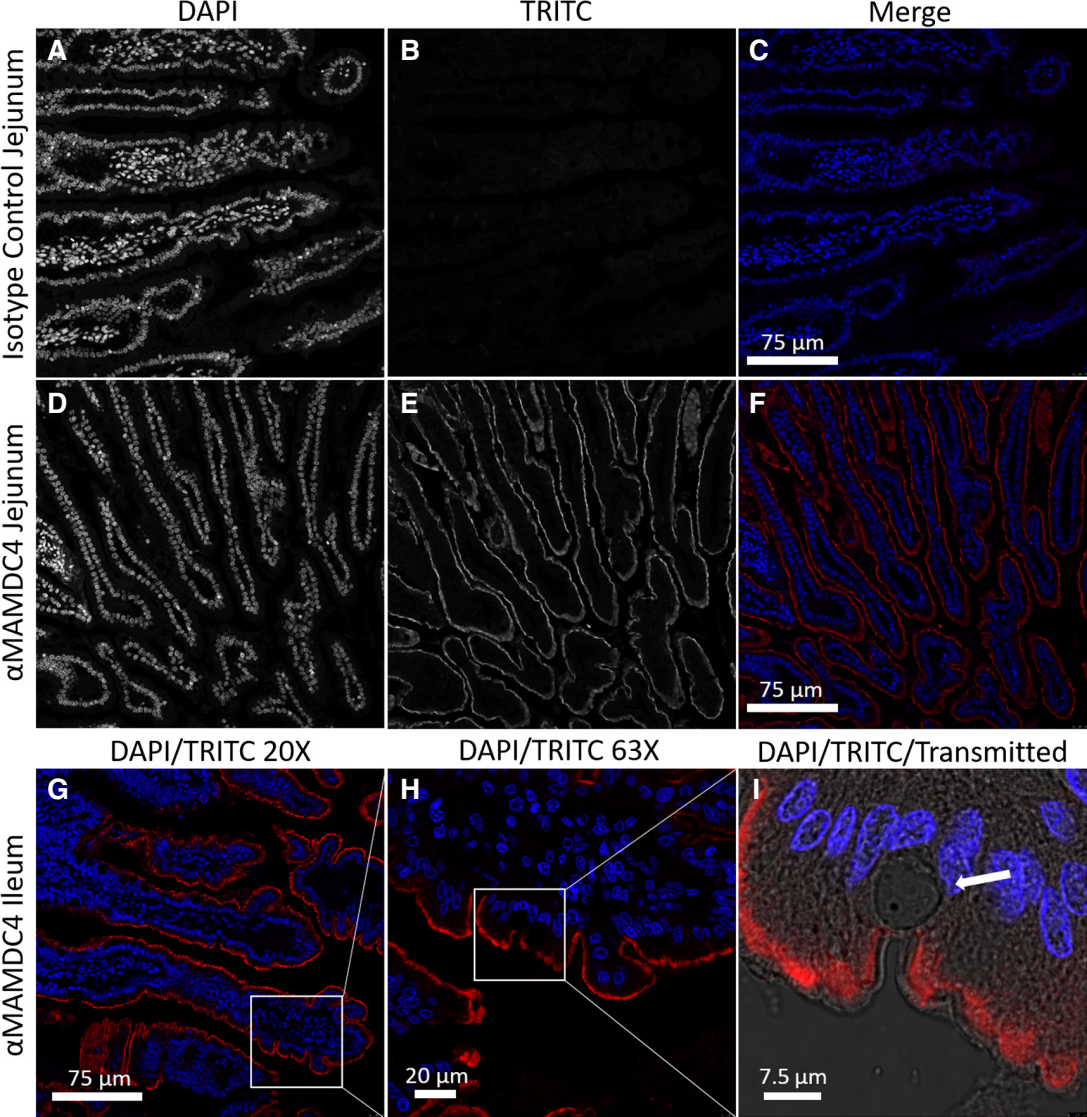
Representative immunohistofluorescence staining of formalin fixed day 3 porcine Jejunum (A–F) and Ileum (G–I), using rabbit isotype control (A–C) and *α* MAMDC4 polyclonal antibody (D–I). Monochrome confocal DAPI (A&D) and TRITC (B&E) images collected at 20× magnification with oil immersion and the resulting colorized and merged images (C&F). Nested images of the Ileum at 20× (G), 63× (H) with 63× with 4× confocal zoom and merged with transmitted (I) arrow indicates a fetal‐type giant vacuole.

We performed immunohistofluorescent analysis on jejunum and ileum tissue sections from piglets at 3 days. The specificity of the anti‐MAMDC4 antibody immunohistofluorescence staining (red) in the jejunum (Fig. 3D–F) in relation to the relevant TRITC‐conjugated rabbit isotype control can be seen in Figure 3A–C. Given this specificity, the merged final image alone is shown for the ileum (Fig. 3G–I) and all remaining figures. The nucleus is stained with DAPI (blue) to shown cellular context. Consistent with previous reports examining the neonatal rat intestine (Wilson et al. 1987), MAMDC4 in the pig gut was identified exclusively on the luminal face of the epithelium (Fig. 3F–I). Furthermore, the protein did not extend to giant vacuoles (Fig. 3I), which are highly abundant in the porcine neonatal intestine and which define “fetal type” vacuolated enterocytes (Smith and Jarvis 1978; Baintner 1994).

**Figure 4:**
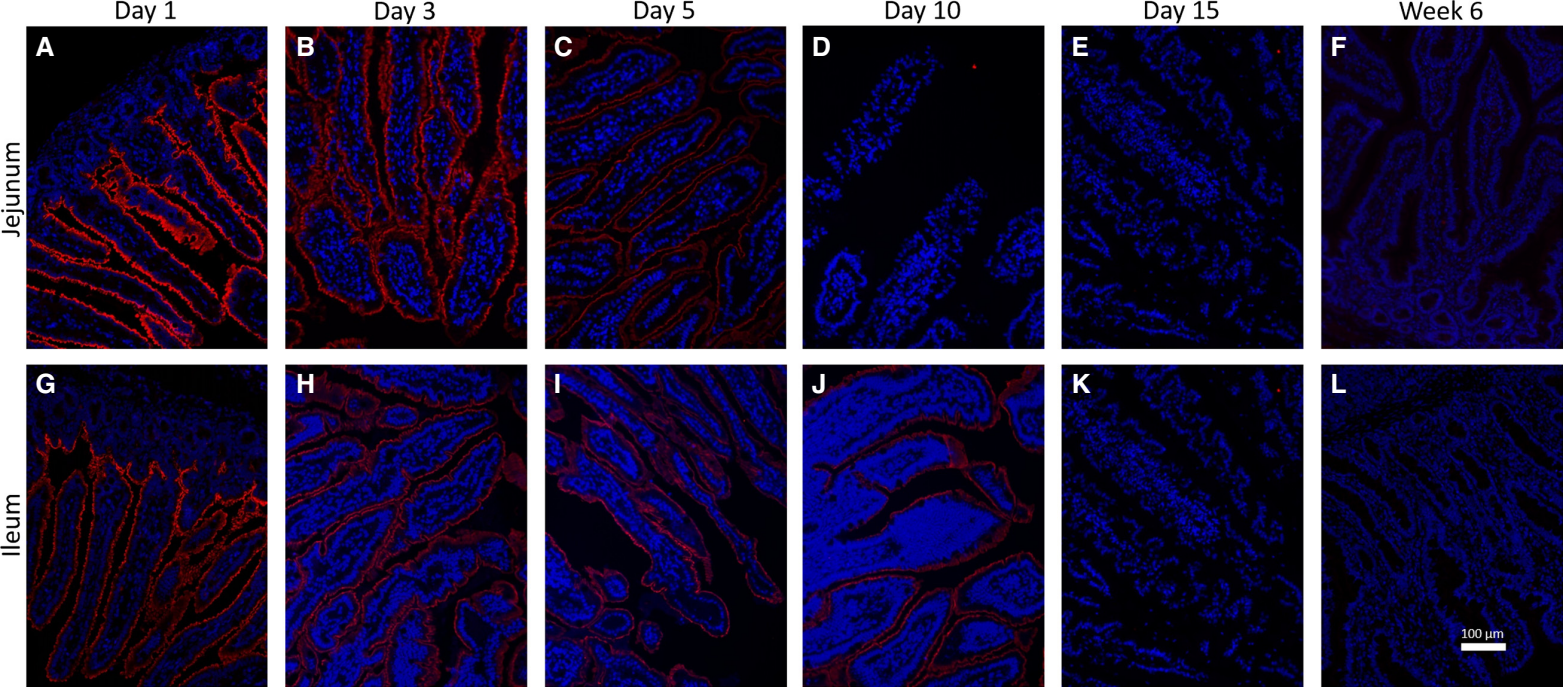
Example 20× standard fluorescent microscopy of immunohistofluorescence staining against MAMDC4 over time course of intestinal development in Jejunum (A–F) and Ileum (G–L) for tissue isolated at Day 1 (A&G) (*N* = 5), Day 3 (B&H) (*N* = 3), Day 5 (C&I) (*N* = 3), Day 10 (D&J) (*N* = 3), Day 15 (E&K) (*N* = 3) and 6 weeks (J&L) (*N* = 5) of age. Staining was conducted on a minimum of two nonconsecutive sections and a minimum of three villi imaged per sample, no outliers were noted.

During the postnatal period, the intestine experiences a while array of anatomical and physiological changes which drastically alter the ratio of epithelial cells to nonepithelial cells. These changes make it difficult to quantitatively evaluate changes to the epithelial proteome. Therefore, to temporally and spatially resolve the postnatal changes in MAMDC4 localization at the cellular level, we next employed immunohisto‐fluorescence to visualize surface expression of MAMDC4 in jejunal and ileal tissues from 1, 3, 5, 10 and 15 days of age as well as postweaning (6 weeks of age) (Jejunum: Fig. 4A–F; Ileum: Fig. 4G–L, respectively). Histology samples from three animals at each time point were examined and representative images are shown in Figure 4. Temporally consistent with the pattern described in the Western blot (Fig. 2), the jejunum and ileum showed robust expression of MAMDC4 on the surface of jejunal (Fig. 4A) and ileal (Fig. 4G) epithelial cells at Day 1 with a steady decrease in MAMDC4 expression over time. A similar loss of MAMDC4 from the surface of adult rat and rabbit intestinal epithelial cells had been reported relative to the neonate (Wilson et al. 1987). In contrast, a recent high‐throughput proteomic analysis of the mouse intestine demonstrated no significant decrease in overall MAMDC4 abundance out to 14 days of age (Hansson et al. 2011). Here we observed that in the neonatal porcine intestine, the decrease in epithelial abundance of MAMDC4 protein expression begins at or prior to birth and the protein is entirely absent by day 10 and 15 in the jejunum and ileum, respectively. This timing is consistent with the reported loss of vacuolated fetal‐type enterocytes from the porcine jejunum after day 7 and in the ileum after day 14, respectively (Skrzypek et al. 2007). Interestingly, the temporal dynamic in protein abundance do not directly correlate with the above described transcriptional changes suggesting MAMDC4 may be regulated, at least in part, at both the transcriptional and translational level.

Other aspects of intestinal development, such as gut “closure” occurs earlier in pigs (Jensen et al. 2001) than in rats and mice (Halliday 1955; Appleby and Catty 1983). In cells that express MAMDC4, we observed no significant change in cellular localization. To correlate this finding with results observed in Figure 2, we suggest that if the lower molecular weight portion of MAMDC4 is internalized from the intestinal lumen, it likely remains associated with the apical endosomal tubules. The comparatively rapid postnatal loss of MAMDC4 suggests it plays a role specific to the unique postnatal intestinal physiology, similar to that observed in other selective transcellular transport mechanisms such as that associate with antibody uptake.

**Figure 5:**
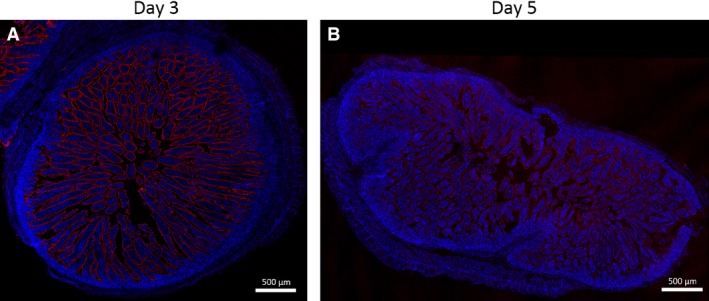
Whole intestinal cross sections of porcine Jejunum at Day 3 (A) and Day 5 (B) following immunohistofluorescence staining against MAMDC4. Images are the result of merged 20× confocal images collected in an overlapping tiled format.

A foundational study involving oral dosing of neonatal pigs (Smith and Jarvis 1978) and other species (Smeaton and Simpson‐Morgan 1985) with [^3^H] thymidine showed cellular division begins in the intestinal crypts and that the epithelial cells migrate up the villi towards the lumen. If indeed fetal‐type enterocytes are shed from the porcine jejunum after day 7 (Skrzypek et al. 2007), we predicted that the postnatal‐derived epithelial cells migrating up the villous from the crypts may show different expression of MAMDC4 over time relative to the fetal enterocytes which are closer to the tips of the villi. To better illustrate the distribution of MAMDC4 along the crypts and villi, we imaged the circumference of the entire jejunal cross‐section from tissues obtained from 3 day old piglets (Fig. 5A) and 5 day old piglets (Fig. 5B). MAMDC4 protein expression on the apical surface can be see along the entire length of the intestinal villi but is absent from the intestinal crypts with a clear qualitative decrease in anti‐MAMDC4 staining intensity along the entire length of the villus over time (Fig. 5B). We do not see that the newer epithelial cells emerging from the crypt and migrating up the villi express lower levels of MAMDC4 than do the fetal‐type enterocytes found nearer the villous tips. This global reduction in MAMDC4 along the length of the villi suggests that loss of MAMDC4 expression is not directly associated with cellular replacement but rather with intercellular regulation.

### Evaluating the role of MAMDC4 in Claudin 1 localization

**Figure 6:**
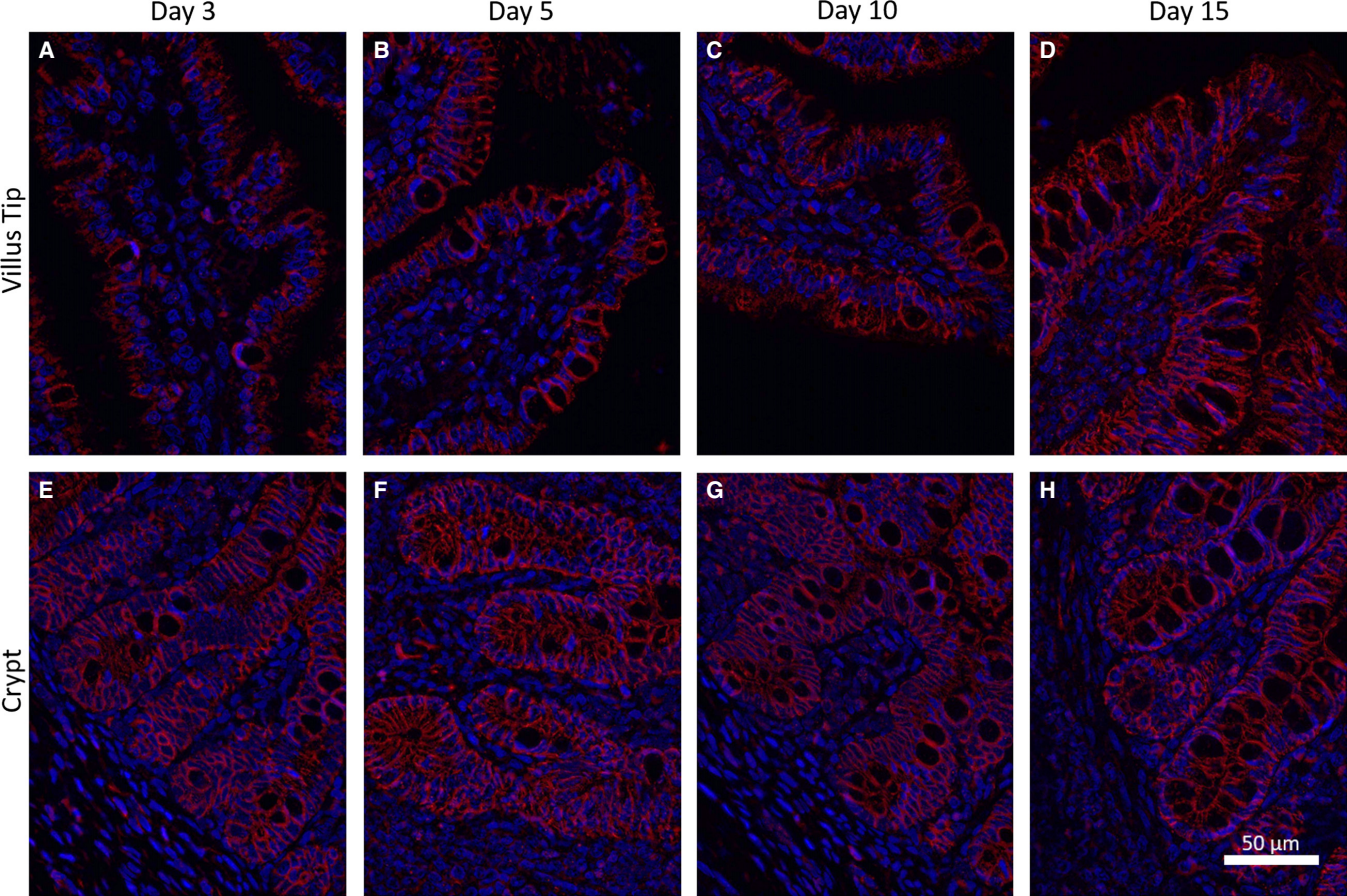
Example 60× standard fluorescent microscopy of immunohistofluorescence staining against CLDN1 at Day 3 (A&E) (*N* = 3), Day 5 (B&F) (*N* = 3), Day 10 (C&G) (*N* = 3) and Day 15 (D&H) (*N* = 3) porcine ileum, showing the tight junction structure at the villus tip (A–D) and crypt (E–H). Staining was conducted on a minimum of two nonconsecutive sections and a minimum of three villi imaged per sample, no outliers were noted.

In vitro studies by others have shown that MAMDC4 may play a role in maintaining transepithelial resistance by regulating the sub‐cellular localization of Occludin and Claudin 1 (CLDN1) to the paracellular tight junction region of the cells (McCarter et al. 2010). To further evaluate the expression and localization of CLDN1 in neonatal piglet gut, we performed immunohistofluorescence on the ileum and focused specifically on the villi from piglets 3 (Fig. 6A), 5 (Fig. 6B), 10 (Fig. 6C) and 15 (Fig. 6D) days of age and we focused specifically on the crypts from piglets 3 (Fig. 6E), 5 (Fig. 6F), 10 (Fig. 6G) and 15 (Fig. 6H) days of age to see if there was a correlation between CLDN1 and MAMDC4 surface expression (Figs. 4 and 5). All intestinal segments from ileum derived from piglets 3–15 days of age showed positive staining for CLDN1 on the luminal surface of the enterocytes lining both the villi and the crypts. No significant differences in apparent CLDN1 protein abundance or localization were noted between days 5 and 15 of development whereas we observed a complete loss of epithelial MAMDC4 expression between ileum obtained at 10 and 15 days of age (Fig. 4I–J). This result would suggest that unlike what has been observed in other systems (McCarter et al. 2010), MAMDC4 may not regulate CLDN1 localization to the tight junction of intestinal epithelial cells. Future studies will clarify whether the porcine intestinal epithelium are capable of maintaining tight junctions in the absence of MAMDC4.

Conversely, the subcellular localization of CLDN1 at day 3 was significantly altered between the epithelium at the base of the villus and in the crypt when compared to that of the villus tip (Fig. 6A and E). Specifically, CLDN1 appears to be localized to the apical surface of the villus epithelial cells (Fig. 6A) but it is localized to the paracellular tight junction region in the crypts (Fig. 6E). This failure to localize CLDN1 to the tight junction occurred when MAMDC4 was highly expressed along the entire length of the villi (Fig. 4A). The transient nature of this relocalization of CLDN1 from the apical aspect of the enterocytes to the paracellular tight junction region over time is consistent with our previous report of abnormal subcellular localization of another tight junction protein, CLDN4, in the intestine of pig during the immediate postnatal period (Pasternak et al. 2015). The temporal and spatial inconsistencies between CLDN1 and MAMDC4 surface expression further support the conclusion that MAMDC4 is not associated with CLDN1 localization in the porcine neonatal intestine.

### MAMDC4 expression in a gnotobiotic model

**Figure 7:**
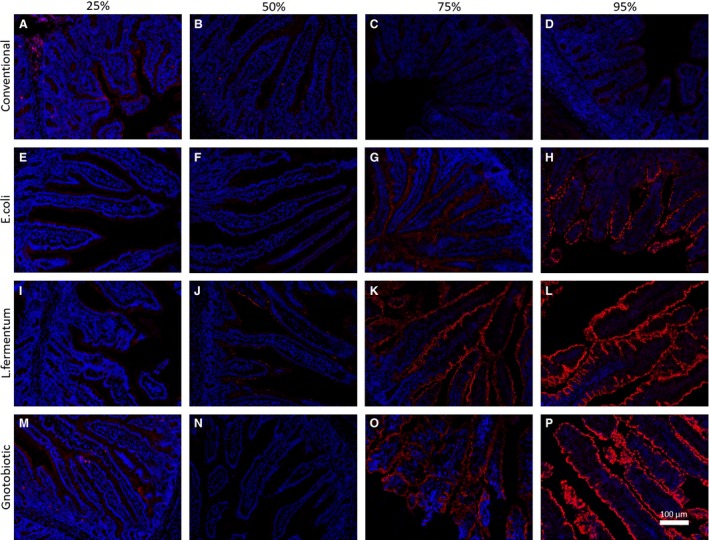
Representative 20× standard fluorescent microscopy of immunohistofluorescence staining against MAMDC4 for intestinal tissue isolated from conventionalized (A–D) (*N* = 4), *E. coli* (E–H) (*N* = 4), *L. fermentum* (I–L) (*N* = 4) and germ‐free (M–P) (*N* = 4) gnotobiotic pigs at 14 days of age. Samples were collected proximally to distally from 25% (A,E,I&M), 50% (B,F,J&N), 75% (C,G,K&O) and 95% (D,H,L&P) of the small intestine. Staining was conducted on a minimum of two nonconsecutive sections and a minimum of three villi imaged per sample, no outliers were noted.

To investigate whether MAMDC4 expression was impacted by colonization, we performed immunohistofluorescence analysis on regions of the small intestine (25%, 50%, 75% and 100% of the length) from CV, EC, LF and GF gnotobiotic pigs at 14 days of age (Fig. 7). Similar to the results observed in the time course of normally reared piglets shown in Fig. 4E and K, the CV group showed no MAMDC4 staining between 50% (Fig. 7B), 75% (Fig. 7C) and 95% (Fig. 7D) of the small intestine at 14 days of age. However, inconsistent low intensity staining of the epithelium was observed in some animals at 25% which may indicate merely background fluorescence (Fig. 7A). Tissues from 25% to 50% of the length of small intestine obtained from EC piglets (Fig. 7E–F), LF piglets (Fig. 7I–J), and GF piglets (Fig. 7M–N) do not show expression of MAMDC4 at 14 days of age. In contrast, positive staining for MAMDC4 was observed in the more distal small intestinal regions (75–95%) for piglets colonized with EC (Fig. 7G–H), LF (Fig. 7K–L) and germ‐free piglets (Fig. 7O–P) despite the tissue being obtained 2 weeks after birth which, in CV or normally reared piglets, has no MAMDC4 expression at this time (Fig. 7C–D). These results suggest that decreased MAMDC4 surface expression in the neonatal intestine is not functionally or temporally‐driven physiological development, but rather the result of bacterial colonization.

That bacterial colonization may impact epithelial cell protein expression is not entirely surprising as variants of this gnotobiotic paradigm have alterations in intestinal morphology (Shirkey et al. 2006), epithelial cell proliferation and apoptosis (Willing and Van Kessel 2007), brush border enzyme activity (Willing and Van Kessel 2009), as well as wide ranging effects to the epithelial transcriptomic (Chowdhury et al. 2007; Meurens et al. 2007) and proteomic (Danielsen et al. 2007) profiles. Despite rigorous analysis, the presently observed effect on MAMDC4 expression (Fig. 1) and abundance (Fig. 2, 3, 4, 5,7) was likely unnoticed by past proteomic (Danielsen et al. 2007) and transcriptomic profiles (Chowdhury et al. 2007; Meurens et al. 2007) for methodological reasons. For example, previous transcriptomic analysis was conducted using the first generation porcine long oligonucleotide array (Zhao et al. 2005) which did not include a probe for the MAMDC4 transcript. The high‐throughput proteomic study focused on the middle portion (50%) of the small intestine in gnotobiotic groups, where the largest effects found in the present study were in the more distal segments of the intestine (75% and 100%) (Danielsen et al. 2007). In addition, the method of protein extraction employed homogenization in TES buffer which likely failed to solubilize membrane‐associated proteins such as MAMDC4. The association of MAMDC4 with bacterial colonization and not tight junction structure is also in keeping with our previous work which showed acute neonate‐specific abnormalities in the subcellular localization of CLDN4 was not associated with bacterial colonization (Pasternak et al. 2015).

Focusing on the gnotobiotic groups (Fig. 7), intense staining for MAMDC4 was observed at 75% and 95% of the length in both GF and LF groups with additional inconsistent staining noted at 50% in only the LF group. In comparison, MAMDC4 staining intensity in the distal segments was substantially reduced in the EC group relative to the LF and GF piglets, particularly at 95%, where the protein was found on the surface of only some villi while entirely absent in others. Staining in the EC group at 75% (Fig. 7G) was more evenly distributed, however the intensity was substantially lower than both LF (Fig. 7K) and GF (Fig. 7O) groups. The intermediate nature of the EC group is consistent with earlier studies which showed alterations in intestinal morphology were also less dramatic compared to CV than those observed in the LF and GF groups (Shirkey et al. 2006). The observed variation between the LF and EC groups in particular suggests that regulation of MAMDC4 is not a result of colonization alone but rather the result of a more specific host‐microbial interaction. This observation is in keeping with the timeline observed in naturally raised piglets where both transcription and translation of MAMDC4 were found to cease at or prior to birth. The reported significant decrease in MAMDC4 expression in the rodent model does not begin until 14 days after birth, however, bacterial colonization still occurs immediately after birth, suggesting that regulation through host microbial interaction may not regulate MAMDC4 expression in all species.

## Conclusions

Our data show that as in other species, MAMDC4 is highly abundant in the piglet neonatal intestinal epithelium. It is down‐regulated both transcriptionally and translationally more rapidly in the postnatal piglet than previously studies species. Although colostral and milk‐borne factors have been reported to play a major role postnatal physiological development (Xu et al. 2000), our data suggest microbial colonization is also critical. In the case of MAMDC4, physiological changes in enterocyte surface expression are mediated, at least in part, by host‐microbial interactions in a bacterial species‐specific manner that does not appear to be solely associated with microbiota‐induced changes in the rate of enterocyte replacement. The apparent lack of effect on the subcellular localization of the tight junction protein CLDN1, suggests that if MAMDC4 plays a role in postnatal transepithelial permeability, it occurs through endocytotic mechanisms rather than via the paracellular route. Furthermore, based on the known specific protein‐binding functions of the other member of the MAM domain‐containing family, we believe MAMDC's role in postnatal endocytotic uptake is likely ligand specific in a fashion similar to that of the neonatal FC receptor (FCGRT) ability to uptake antibody. Although the role of MAMDC4 in early maturation of the postnatal intestinal epithelium remains ambiguous, further work should consider the influence of postnatal microbial succession as an important regulatory factor.

## Conflict of Interest

None declared.
